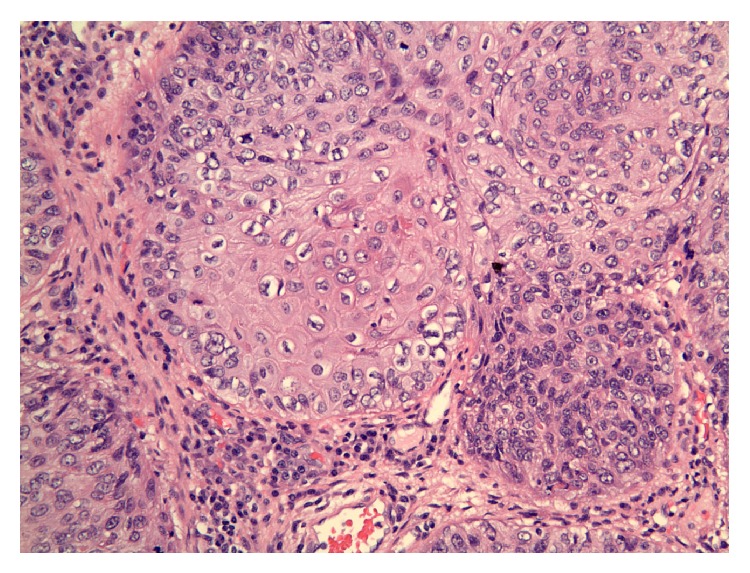# A Gigantic Anogenital Lesion: Buschke-Lowenstein Tumor

**DOI:** 10.1155/2014/650714

**Published:** 2014-11-06

**Authors:** Rikinder Sandhu, Zaw Min, Nitin Bhanot

**Affiliations:** Department of Medicine, Allegheny General Hospital, 420 East North Avenue, Allegheny Health Network, Pittsburgh, PA 15212, USA

## Abstract

Buschke-Lowenstein tumor is a relatively rare sexually transmitted disease. It is a neoplasm of the anogenital region which has benign appearance on histopathology but is locally destructive. It carries a high recurrence rate and a significant potential for malignant transformation. Human papilloma virus has been implicated as an etiologic agent for this tumor. Since this disease is rare and no controlled studies exist, radical excision of this anogenital lesion is generally recommended as the first line therapy and close vigilance and followup are essential. We have discussed an overview of etiopathogenesis, clinical presentation, diagnosis, and management of this uncommonly encountered disease.

## 1. Case Presentation

A 65-year-old man presented to the hospital with 1-week history of bloody and foul smelling discharge from his groin. The patient had initially noticed a small lesion of approximate dimension of a pea in his right inguinal area about 20 years ago. Since the lesion did not bother him at that time, he did not seek medical care until when he started to notice blood oozing from it. The lesion had increased in size multifold over the years and had extended into the left groin and the perineal region. It was not painful, but caused some discomfort mainly during sitting. He reported no fevers, loss of weight, urinary symptoms, or difficulty in defecation and denied any other skin lesions elsewhere. All other reviews of systems were essentially unremarkable. He was in a monogamous relationship with his wife who had passed away 10 years prior to presentation. He denied previous promiscuous behavior.

**Figure 1:**
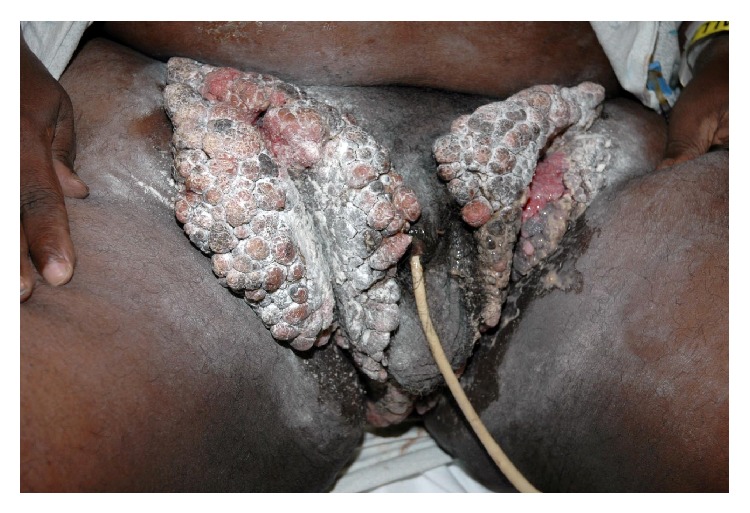


On examination, patient was afebrile and hemodynamically stable. An extensive, foul-smelling cauliflower-shaped lesion was apparent in the inguinal regions bilaterally. It measured around 15 cm in width in each groin along the maximum dimension, and about 40 cm in length, extending from the waist line through the groin into the intergluteal cleft towards the rectum (Figure 1). It had also involved the lateral aspects and base of the scrotum. The lesion was not tender. Remainder of the physical examination was normal. Laboratory studies revealed leukocytosis (white blood cell count 15,300 per mm^3^), hemoglobin 14.3 gm/dL, and platelet count 284,000 per mm^3^. Renal and liver function tests were normal.

Human immunodeficiency virus and rapid plasma reagin serological tests were nonreactive. Urine analysis and blood cultures were unremarkable. Contrast enhanced computed tomography of chest, abdomen, and pelvis was done that did not reveal any regional lymph node involvement or evidence of distant metastasis.

**Figure 2:**
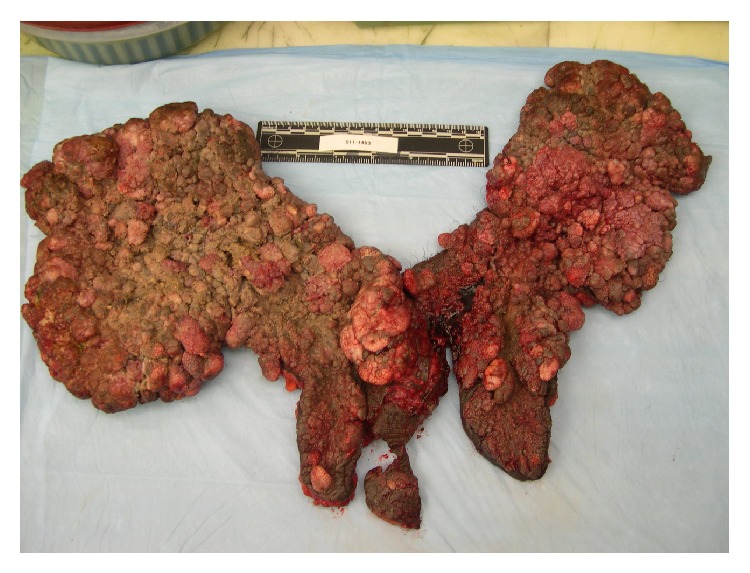


**Figure 3:**
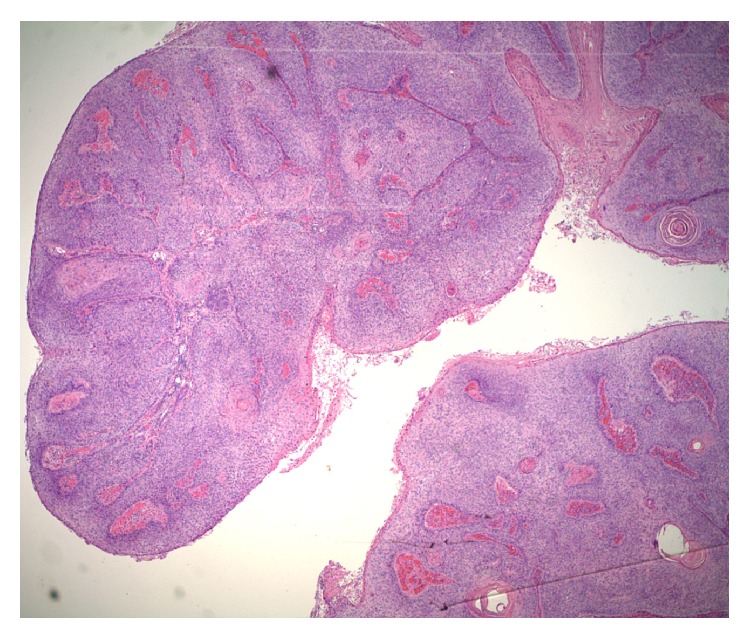


Based on the clinical presentation, a diagnosis of giant condyloma acuminatum of Buschke-Lowenstein was suspected. The lesion was radically excised and the entire specimen was sent for gross pathological examination (Figure 2) and histopathology (Figures 3 and 4). Grossly, the tumor appeared as fungating, exophytic, cauliflower-shaped like mass. It was hard in consistency and gave the look of a bulky tumor. On histopathology, there was evidence of epidermal hyperplasia, hyperkeratosis, and papillomatosis (Figure 3). Koilocytes (vacuolated cells with clear cytoplasm and perinuclear halo), a result of infection from HPV, were seen on higher magnification (Figure 4). There were no histopathologic features of malignant transformation.

A final diagnosis of condyloma acuminata of BLT (superficially invasive verrucous carcinoma with pathological staging T1) was made. The patient has not had a recurrence of the tumor for 3 years from the time of radical excision.

## 2. Discussion

Giant condyloma acuminata was first described by Buschke and Loewenstein in 1925 in the penis and they named it “condyloma acuminate carcinoma-like” [1]. Since then, it has also been reported in the anorectal and perineal regions [2]. The vulva is the predominant location in females [3]. While the characteristic feature of Buschke-Lowenstein tumor (BLT) is benign appearance on histopathology, the lesion has locally destructive behaviour and may undergo malignant transformation [2, 4]. Therefore, some authors support the hypothesis that BLT is an intermediary lesion between condyloma acuminata and squamous cell carcinoma [4]. However, others and probably a majority of them, equate it to verrucous carcinoma (a well-differentiated variety of squamous cell carcinoma) of the anogenital region [3].

It is a sexually transmitted disease with an estimated incidence of about 0.1% in the general population [3]. Human papilloma virus (HPV) has been linked to the etiopathogenesis of BLT [3]. HPV DNA types 6 and 11 have been most commonly recovered from pathological specimens of BLT, suggesting a pathogenic role [2].

To confirm histopathologically, deeper tissue must be biopsied to ensure that no malignant cytological characteristics are missed in superficially biopsied specimens [3]. Radical excision of the entire lesion is suggested because it serves the dual purpose of making the diagnosis and helping therapeutically with the highest chances of cure [3].

While a variety of treatment modalities (surgery, chemotherapy (systemic and intralesional), carbon dioxide laser therapy, and photodynamic therapy) have been used for the treatment of BLT, wide surgical excision by Mohs technique is recommended as the most important therapeutic intervention [2]. Since this is a rare disease and no robust controlled studies have been conducted, no standardized management strategies exist. Chemoradiation has been recommended in cases when malignant transformation may occur [3]. Intra-arterial chemotherapy with agents such as methotrexate has also been successfully utilized in verrucous carcinoma of different parts of the body, including the anogenital region [5, 6]. This modality may be used as neoadjuvant therapy before surgical intervention and in certain cases may obviate the need for surgery [5, 6]. Radiation therapy alone has generally been discouraged because of potential risk of transformation into anaplastic carcinoma [7].

Since radical excision was achieved with peripheral and deep margins free of tumor and no regional lymphadenopathy was noted, the oncologist did not recommend additional modalities such as chemotherapy in our patient. Vigilant and prolonged surveillance was suggested. This is important because this tumor has been reported to have a significant rate of recurrence (66%) and malignant transformation (56%) with an overall mortality of 20% [8]. The tumor may also form abscesses and fistulae in the perianal region [9].

Since all lesions of BLT initially start as condyloma acuminata and progress over many years, this entity is likely preventable to an extent by vaccination against certain strains of HPV and seeking timely medical attention. Our case adds to this relatively rare disease and highlights the importance of timely detection, aggressive management, and close surveillance to improve patient outcomes.

## Figures and Tables

**Figure 4:**